# Normal form from biological motion despite impaired ventral stream function

**DOI:** 10.1016/j.neuropsychologia.2011.01.009

**Published:** 2011-04

**Authors:** S. Gilaie-Dotan, S. Bentin, M. Harel, G. Rees, A.P. Saygin

**Affiliations:** a Institute of Cognitive Neuroscience, University College London, London, UK; b Wellcome Trust Centre for Neuroimaging, University College London, London, UK; c Department of Psychology, Hebrew University of Jerusalem, Jerusalem, Israel; d Interdisciplinary Center for Neural Computation, Hebrew University of Jerusalem, Jerusalem, Israel; e Department of Neurobiology, Weizmann Institute of Science, Rehovot, Israel; f Department of Cognitive Science and Neuroscience Program, University of California San Diego, 9500 Gilman Drive, San Diego, CA 92093-0515, USA

**Keywords:** Biological motion, Form agnosia, Form from motion, Point-light displays, Ventral visual stream

## Abstract

We explored the extent to which biological motion perception depends on ventral stream integration by studying LG, an unusual case of developmental visual agnosia. LG has significant ventral stream processing deficits but no discernable structural cortical abnormality. LG's intermediate visual areas and object-sensitive regions exhibit abnormal activation during visual object perception, in contrast to area V5/MT+ which responds normally to visual motion (Gilaie-Dotan, Perry, Bonneh, Malach, & Bentin, 2009). Here, in three studies we used point light displays, which require visual integration, in adaptive threshold experiments to examine LG's ability to detect form from biological and non-biological motion cues. LG's ability to detect and discriminate form from biological motion was similar to healthy controls. In contrast, he was significantly deficient in processing form from non-biological motion. Thus, LG can rely on biological motion cues to perceive human forms, but is considerably impaired in extracting form from non-biological motion. Finally, we found that while LG viewed biological motion, activity in a network of brain regions associated with processing biological motion was functionally correlated with his V5/MT+ activity, indicating that normal inputs from V5/MT+ might suffice to activate his action perception system. These results indicate that processing of biologically moving form can dissociate from other form processing in the ventral pathway. Furthermore, the present results indicate that integrative ventral stream processing is necessary for uncompromised processing of non-biological form from motion.

## Research highlights

▶ Normal biological motion processing can exist independently from form processing. ▶ Intact ventral stream processing is not necessary for biological form-from-motion. ▶ Proper ventral stream processing is necessary for non-biological form-from-motion. ▶ Normal visual inputs from V5/MT+ can suffice to activate the action perception system. ▶ Biological motion can be processed successfully even with compromised ventral stream.

## 1 Introduction

Visual perception of body movements of other animate entities is fundamental to our survival and well-being. Perception of biological motion is a crucial component of this ubiquitous and important process (Blake & Shiffrar, 2007). One frequently exploited technique in the study of biological motion is point-light displays (Johansson, 1973). Animated point-light displays of human figures comprise about a dozen markers attached to the limbs of a person and when in motion, provide compelling demonstration of biological form from motion. Even though they comprise only a few point lights, these stimuli can depict a person's body movements vividly, conveying detailed information such as gender, identity, and emotions (e.g., Dittrich, Troscianko, Lea, & Morgan, 1996; Perry, Troje, & Bentin, 2010; Pollick, Paterson, Bruderlin, & Sanford, 2001; Troje, 2002). Biological motion perception is supported by a network of brain areas, including temporal, frontal and parietal cortical regions (Blake & Shiffrar, 2007; Grossman & Blake, 2002; Puce & Perrett, 2003; Rizzolatti & Sinigaglia, 2010; Saygin, 2007; Saygin, Wilson, Hagler, Bates, & Sereno, 2004), here referred to as the “Action Perception System” (APS).

Even with point-light displays, biological motion involves form processing as well as motion processing *per se*. This raises the question of whether biological motion perception and form processing interact or are independent. The involvement of form processing in biological motion perception has indeed been supported by several psychophysical, neuroimaging and neurophysiological studies (Beintema, Georg, & Lappe, 2006; Bruce, Desimone, & Gross, 1981; Grossman & Blake, 2002; Lange & Lappe, 2006; Neri, Morrone, & Burr, 1998; Vangeneugden, Pollick, & Vogels, 2009). Others have highlighted the importance of dynamic cues in biological motion perception (Cavanagh, Labianca, & Thornton, 2001; Giese & Poggio, 2003; Thurman & Grossman, 2008).

**Fig. 1:**
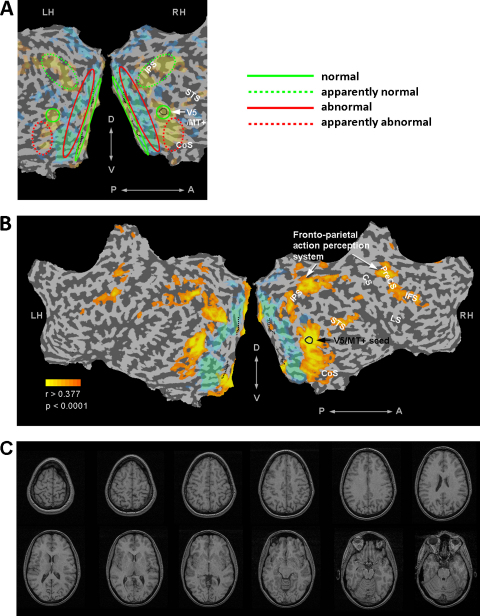
Functional organization of LG's visual cortex on flattened cortical maps. (A) Delineation of LG's visual system organization (see Gilaie-Dotan et al., 2009) displaying normal V1 and V5/MT+ response patterns (indicated by green contours), abnormal deactivations in intermediate visual regions (red contours). LG's dorsal stream appeared normal (dotted green). His ventral stream lateral occipital areas were activated above normal, but did not display the expected sensitivity to object stimuli (dotted red). (B) Functional connectivity of LG's right V5/MT+ (delineated in black contour) to the fronto-parietal nodes of the action perception system (APS) during viewing movie clips that included biological motion (see Section 6.1). Yellow to orange patches display regions that were significantly correlated with LG's right V5/MT+ activity while he was watching the video clips (*r* >  = 0.377, *p*_corrected_ < = 0.0001). LG's V5/MT+ functional connectivity pattern to the APS resembles the one seen in the normal brain (e.g., Saygin et al., 2004), in contrast to his intermediate visual cortical areas (shaded in turquoise). (C) Structural images of LG's brain. No discernable cortical abnormality was detected (Gilaie-Dotan et al., 2009). IPS – intraparietal sulcus, PreCS – precentral sulcus, IFS – inferior frontal sulcus, STS – superior temporal sulcus, LS – lateral sulcus, CS – central sulcus, RH – right hemisphere, LH – left hemisphere, P – posterior, A – anterior, D – dorsal, V – ventral. (For interpretation of the references to color in this figure legend, the reader is referred to the web version of this article.)

In the present study, we used point-light displays to investigate whether biological motion perception depended on form processing in LG, an individual known to have developmental form processing deficits but who reports normal motion perception. Behaviourally, LG has problems integrating visual stimuli into coherent forms. In contrast, based on his introspection, he recognizes people by the way they walk, suggesting that his biological motion perception is not deficient (Table 1). In a recent fMRI study, we found that the activity in LG's visual cortex associated with visual stimulation was consistent with such a dissociation between motion and form processing (Gilaie-Dotan, Perry, Bonneh, Malach, & Bentin, 2009). That earlier study found that abnormal activity in LG's intermediate visual regions leads to impaired sensitivity to objects in higher-level object sensitive areas (e.g., LO, see Fig. 1A, red dotted regions), while responses in motion sensitive area V5/MT+ to visual motion were normal (see Fig. 1A, V5/MT+ denoted in green).

In brief, LG displays apparently normal motion sensitivity, yet exhibits form processing impairments. We therefore reasoned that examining his biological form from motion perception using point-light displays might shed light on the dependence of biological motion processing on form integration. In addition, we also tested LG's non-biological form from motion (Hiris, 2007; Saygin, Cook, & Blakemore, 2010) allowing us to examine whether recognition of moving forms by different motion types displayed different dependencies on form integration and ventral stream processing.

We tested LG and compared his performance with that of age-matched healthy controls in three experiments that measured perceptual thresholds for form from motion perception using biological motion (in Experiments 1 and 2) and non-biological motion (in Experiments 2 and 3). We have used the paradigms of Experiments 1 and 2 successfully in previous studies with stroke patients (Saygin, 2007) and autism spectrum conditions (Saygin et al., 2010). The paradigm of Experiment 3 was also based on a previously published technique (Singer & Sheinberg, 2008).

We also wanted to examine the functional integrity of the APS in LG's brain, given that there are sparse and abnormal visual inputs from LG's V2/V3 and ventral stream (Gilaie-Dotan et al., 2009). Since a primary source of input into the APS is the motion sensitive visual region V5/MT+, we assessed whether the activity in the APS regions was functionally correlated to LG's right V5/MT+ activity during natural viewing of biological and non-biological motion movie clips.

## 2 General methods

### 2.1 Participants

LG was 23 years old when tested on Experiments 1 and 2; and 24 years old when tested on Experiment 3. Thirteen age-matched control participants were tested in Experiment 1 (mean age 20.42 ± 1.08 (S.D.)), 21 in Experiment 2 (mean age 23.29 ± 4.88), and 8 in Experiment 3 (mean age 22.62 ± 2.45). Approximately half of the control participants were women. All control participants had normal or corrected to normal vision and no history of neurological disorders. LG was tested in his home. Control participants were tested at the Institute for Cognitive Neuroscience, University College London. All participants gave written informed consent and the experiments were approved by local ethics committee (University College London).

## 3 Experiment 1

**Fig. 2:**
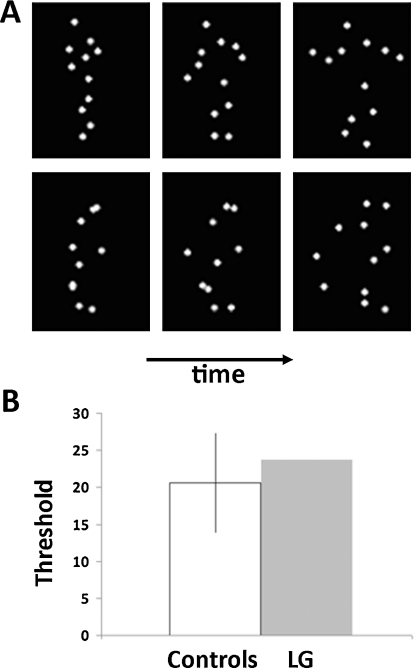
Experiment 1: paradigm and results. (A) Example frames from the stimuli used in the experiment (Saygin, 2007). Top: Three still frames from one of the human motions used (see Supplementary Materials for animations). Bottom: Scrambled version of the human motion (see text). Human motion and its scrambled version were presented simultaneously on either side of the screen and participants had to determine the side in which the human motion was presented, without time restrictions. Noise points were added in an adaptive manner to both animations to reach 82% accuracy. (B) Results showing the estimated thresholds (in number of noise points) for LG (gray) and 13 age-matched controls (white). LG's performance was within the normal range. Error bar indicates one standard deviation.

In this experiment we assessed the ability of LG to process form from biological motion. On each trial, LG and control participants were presented with two point-light displays presented simultaneously and were instructed to decide which of the two displays contained an animation of an upright human figure performing one of seven actions (see Section 3.1.1, Fig. 2A, Supplementary Figs. 1–3; Saygin, 2007).

### 3.1 Experiment 1: methods

#### 3.1.1 Experiment 1: stimuli

Biological motion animations were created by videotaping an actor performing various activities, and encoding only the joint positions in digitized videos (Ahlstrom, Blake, & Ahlstrom, 1997). In the videos, the joints were represented by 12 small white points against a black background (Fig. 2A; for an animated example, see Supplementary Figs. 2 and 3). The biological motion animations depicted one of seven actions: walking, jogging, throwing, underarm throwing (bowling), stepping up, a high kick into the air, and a lower kick. Each animation consisted of 20 distinct frames and was displayed for 0.5 s (16.5 ms interframe interval, 60 Hz). The final frame then remained visible for 0.3 s, after which the animation looped from the beginning. Since a joint could become occluded by other body parts during an action, some points could at times become briefly invisible.

For each of the seven biological motion animations, a matched spatially scrambled animation was created. This was done by scrambling the starting positions of the 12 points while keeping the moving trajectories of each point unchanged. Hence, the scrambled animations contained the same local motion information as the biological motion animations, but without the global form of the latter. The starting positions of the scrambled points were chosen randomly within a region such that the total area encompassed by the scrambled animation was similar to that of the biological animation.

During each trial, the displays of both biological motion stimuli and their scrambled counterparts had additional moving noise points randomly superimposed on them. The moving trajectories of these noise points were generated in the same way as of the scrambled animations. The task became more difficult with increasing number of noise points. The number of noise points was manipulated in an adaptive procedure (see below).

Each animation subtended approximately 4 × 6° (width × height) visual angle when viewed from 55 cm. The total area occupied by each point-light display (comprising the animation plus the noise points) was approximately 7° of visual angle in diameter. On each trial, the two point-light displays (one containing the biological motion animation, the other containing its scrambled counterpart) were displayed to the left and right of the centre of the screen respectively (centred at approximately 9° from the centre of the screen), their vertical centres horizontally aligned (see Supplementary Fig. 1). Stimuli were presented and responses recorded using MATLAB (Mathworks, Natick, MA, USA) and the Psychophysics Toolbox V2.54 (Brainard, 1997; Pelli, 1997).

#### 3.1.2 Experiment 1: procedure

Participants were familiarized with the seven types of biological motion animations before the experiment started. At this stage, each animation was displayed on the screen (without any noise points superimposed) and the participants described verbally what they perceived.

Following the familiarization phase, sensitivity to biological motion was assessed using a 2-AFC experimental design. Two point-light displays were displayed in each trial (see details above), one containing a biological motion animation (one of the seven human actions, see above) and the other containing its scrambled counterpart. The side of the biological motion animation was randomly determined on each trial. Participants were instructed to press one of two keys on the keyboard indicating the side for which the animation represented ‘a person’. The animations were repeated continuously until a response was given. Participants were not required to fixate (e.g., at the centre of the screen); instead, they were allowed to look at the stimuli as they pleased.

To yield a psychometric measure of performance, we varied the number of noise points in each trial using a Bayesian adaptive procedure that efficiently estimated the level of noise at which a participant performed at a desired level of accuracy (QUEST, Watson & Pelli, 1983). After the familiarization phase, control participants performed total of 118 adaptive trials, and an accuracy threshold of 82% was estimated for each participant using the mean of the posterior probability density function. These trials were presented in two equal blocks separated by a 10-s rest period (in fact, since the task was not timed, subjects could take additional breaks at any time). Control participants completed one run of the experiment. LG completed two runs of the experiment, each with 73 adaptive trials, with a rest period of 10 s after 40 trials in each run.

#### 3.1.3 Experiment 1: data analysis

The perceptual threshold was defined as the estimated number of noise points that allowed each participant to perform at the pre-determined accuracy level, as described above. Thresholds from multiple runs were averaged. We considered LG's performance to be significantly different from controls if it differed by more than two standard deviations from the mean of the controls’ performance. These differences in thresholds between LG and controls were further confirmed by using established statistical procedures to compare between single cases and controls (Corballis, 2009; Crawford, Garthwaite, & Howell, 2009).

### 3.2 Experiment 1: results and interim discussion

This experiment tested LG's sensitivity to point-light biological motion (Saygin, 2007). During familiarization, neither LG, nor any of the controls had trouble recognizing the movements depicted by the biological motion point-light displays (see Fig. 2A; Supplementary Figs. 2 and 3). LG was immediately able to report the correct movements presented during familiarization. The noise point thresholds for the main 2AFC part of the experiment are shown in Fig. 2B. LG's performance was clearly within the range of the controls (LG: 23.66, 0.45 S.D. above controls’ mean (20.58 ± 6.80 (S.D.)), see Fig. 2B). Statistical analysis confirmed this (*t*(12)_Crawford_ = 0.436, *p* = 0.67; *t*(12)_Corballis_ = 0.452, *p* = 0.659). This pattern was not influenced by learning as it held even when we examined performance on the first session separately (LG: 20.52, 0.16 S.D. above controls’ mean for first session 19.53 ± 6.26 (S.D), *t*(12)_Crawford_ = 0.151, *p* = 0.88; *t*(12)_Corballis_ = 0.157, *p* = 0.877).

The results of this experiment indicate that LG was able to detect human figures normally based on their characteristic biological motion. Thus, the functional impairment in his ventral system did not appear to interfere with his ability to perceive form from biological motion, even when integration was essential for the task.

## 4 Experiment 2

This experiment aimed to assess LG's ability to perceive form from biological or non-biological motion (Hiris, 2007; Saygin et al., 2010). A single point-light animation was displayed on each trial, and participants were instructed to determine whether the target (defined either by biological motion or non-biological translational motion) was moving to the right or to the left. In the biological motion condition the target was an animation depicting a person walking, which featured a recognizable, familiar biological form. In the two non-biological motion conditions, the point-light animated target formed either a familiar form (a rectangle), or an unfamiliar form (see Section 4.1.1 for details).

### 4.1 Experiment 2: methods

#### 4.1.1 Experiment 2: stimuli

**Fig. 3:**
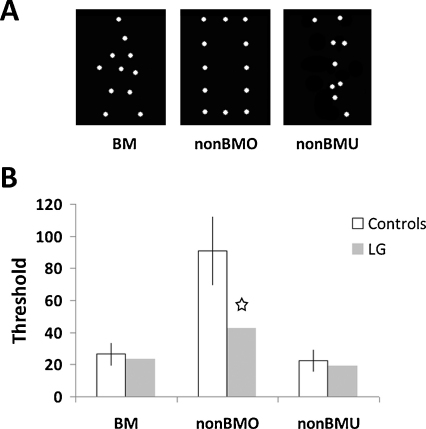
Experiment 2: paradigm and results. (A) Examples (snapshots) of the targets from the three conditions. BM condition comprised of a point-light walker, nonBMO comprised a translating rectangle, and the nonBMU comprised a translating unstructured object. Participants had to determine whether the target was moving to the right or to the left while it was masked by an adaptive amount of noise points moving to both directions (see text). (B) Results showing the estimated thresholds (in number of noise points) for LG (gray) and 21 age-matched controls (white) for each of the conditions. LG's performance for biological motion (BM) was within the normal range (left), for the structured form translation (nonBMO) he was significantly below controls (middle, denoted by an asterisk), and for the unstructured form (nonBMU) within controls’ range (right). Error bars indicate one standard deviation.

This experiment comprised three conditions, each featuring a different type of point light display, all composed of white points presented on a black background. Still frames depicting the three types of stimuli for each of the three experimental conditions are shown in Fig. 3A. The biological motion (BM) point-light animation was identical to the walking figure from Experiment 1 (for an animation demo see Supplementary Fig. 3). The walking movement did not include translation (as if walking on a treadmill), like in most studies on biological motion. The direction that the walker faced (right or left) was determined randomly on each trial. The familiar non-biological motion object (nonBMO) was a rectangle made of equidistant points and translated at 0.5 pixels/frame (at 60 Hz) to the right or to the left (again determined randomly). The unfamiliar non-biological motion object (nonBMU) was an unstructured form translating as in the nonBMO condition. The nonBMU was obtained by taking a single frame from the biological motion animation and presenting it upside-down. Such inverted point light displays are typically perceived as a set of random points, and inversion greatly disrupts the percept of a biological figure (Pavlova & Sokolov, 2003; Saygin, Driver, & de Sa, 2008; Sumi, 1984).

As in Experiment 1, a variable number of noise points with similar motion trajectories as the targets were also presented on each trial. The initial spatial location of the noise points was determined randomly. In the biological motion (BM) condition, each noise point had the motion trajectory of one of the points from the target biological motion animation. The noise points in the non-biological motion conditions (nonBMO and nonBMU) translated horizontally. A number of the noise points (equal to those of the target) always moved in the opposite direction to the target point light animation, so that it would not be possible to determine the direction of target movement simply by summation of the overall movement direction in the display. The rest of the noise points moved either to the left or to the right randomly.

The target point-light displays subtended approximately 4 × 8° visual angle when viewed from 55 cm while the region populated by the target point-light display and the noise points together was approximately 8 × 12° visual angle. On each trial, the target point-light display was presented at a randomly jittered location within a 2.2° radius from the centre of the screen. Stimuli were presented and responses recorded using Matlab (Mathworks, Natick, MA, USA) and the Psychophysics Toolbox V2.54 (Brainard, 1997; Pelli, 1997).

#### 4.1.2 Experiment 2: procedure

For each condition the experiment started with a practice block, which included up to 20 trials of that condition, with a range of predetermined number of noise points (ranging from 0 to 70). The practice was followed by the main experimental block for that condition, which included 60 adaptive trials, beginning with 20 noise points. To measure discrimination thresholds for the direction of motion in each condition, we used the same Bayesian adaptive paradigm as in Experiment 1 (QUEST). The number of noise points was varied from trial to trial, and we estimated the number of noise points at which each participant performed at 75% accuracy (Watson & Pelli, 1983). A 10-s break followed trial 36 in each block, and additional rest was allowed between blocks. Each block lasted between 3 and 4 min. Participants completed three blocks of each condition.

Each trial started with a white fixation cross displayed at the centre of the screen for 750 ms, after which the point-light displays were presented along with noise points (see more details above). Participants pressed one of two keys to indicate the perceived movement direction of the target point-light display (right or left). The task became more difficult with increasing number of noise points. If no response was given within 2000 ms from the end of the stimulus presentation, the trial was terminated and an incorrect response was used in the QUEST algorithm. After each response, a visual feedback cue appeared for 750 ms (green fixation cross for correct and red for incorrect).

#### 4.1.3 Experiment 2: data analysis

For each experimental condition thresholds were calculated for LG and for each of the control participants and data were analyzed as in Experiment 1.

### 4.2 Experiment 2: results and interim discussion

As in Experiment 1, LG's biological motion (BM) detection was well within the controls’ range in Experiment 2 (LG: 23.43, 0.43 S.D. from the control mean, which was 26.53 ± 7.16 (S.D.); *t*(20)_Crawford_ = −0.423, *p* = 0.676, *t*(20)_Corballis_ = −0.432, *p* = 0.669; see Fig. 3B). In contrast, LG performed significantly worse than controls in the non-biological structured object (nonBMO) condition (LG: 42.47, 2.26 S.D. below controls’ average of 91.06 ± 21.52; *t*(20)_Crawford_ = −2.21, *p* = 0.035, *t*(20)_Corballis_ = −2.26, *p* = 0.039). Finally, with the unstructured stimuli (nonBMU), both LG and controls performed equally poorly (LG at 19.33, 0.46 S.D. from controls mean 22.53 ± 7.02; *t*(20)_Crawford_ = −0.45, *p* = 0.66, *t*(20)_Corballis_ = −0.46, *p* = 0.653).

The important comparisons related to our research question (whether LG's form from motion perception was normal) are found in the within-condition comparisons of LG to controls. Consistent with the findings of Experiment 1, comparing the performance of LG and controls indicated that LG was indeed able to process biological motion as well as controls, now confirmed with a second task (direction discrimination). However, his ability to discriminate the direction of a moving structured object defined by non-biological form from motion was significantly worse than that of controls. Between-condition comparisons revealed that thresholds differed significantly between conditions (all pairwise differences were significant, *p* < 0.05), broadly consistent with findings by Hiris (2007). However, the raw thresholds of the biological motion (BM) and the non-biological motion conditions (nonBMO and nonBMU) are not comparable since form from motion is conveyed very differently between the two types of motion. As for the non-biological motion conditions, even though the thresholds of non-biological motion conditions (nonBMO and nonBMU) were significantly different (*p* < 10^−12^), there was a strong and significant correlation between them (*r* = 0.58; *r*^2^ = 0.32, *t*(19) = 3.14, *p* = 0.005), indicating that these are likely to be processed by some joint mechanisms. There was a weaker correlation between biological motion (BM) thresholds and the non-biological structured object (nonBMO, rectangle) thresholds (*r* = 0.414; *r*^2^ = 0.17, *t*(19) = 1.99, *p* = 0.062).

LG appears to have limited ability to utilize form cues in form from non-biological motion perception. For control participants, thresholds (Experiment 2) were notably higher in the non-biological structured object condition (nonBMO) compared with the non-biological unstructured condition (nonBMU). In contrast, LG showed a more modest increase in noise point threshold for the structured object, compared with the unstructured object, likely because he could not rely on an intact ventral stream to fully take advantage of the form information that makes the non-biological structured object condition (nonBMO) much easier for controls. We hypothesize that controls, with normal visual integration mechanisms (Lerner, Hendler, & Malach, 2002), can utilize integration mechanisms in the ventral stream to improve their performance when the moving object has a coherent form. The rectangle stimulus used in this condition (nonBMO condition) conveyed a strong Gestalt, which even LG was able to use. However when the rectangle was masked with noise points the integration process became more difficult. For the unstructured object condition (nonBMU), controls, as well as LG, were not able to use integration benefits since the form did not convey a strong Gestalt.

The apparent dissociation between LG's normal performance on form from biological motion compared to his impaired performance on the form from non-biological motion might be due to the biological aspect of the motion, and that there may be unique pathways supporting biological motion processing. However, there are additional differences between these conditions. LG's ability to successfully recognize biological figures may stem from the fact that these stimuli have an induced object-typical motion, rather than from the biological nature of that induced motion *per se*. Humans have a typical, characteristic motion, whereas rectangles do not. It is possible that LG was more familiar with the object-typical biological motion in the biological motion (BM) condition than with the somewhat arbitrary pairing of rectangular form and linear motion we used in the non-biological structured object (nonBMO) condition (though presumably, so were control participants (Cavanagh et al., 2001)). Another possible distinction between these conditions could be related to the dimensionality of the induced percept. The rectangle in the non-biological structured object (nonBMO) condition was a 2D shape and the translating motion did not induce any additional depth cues. The biologically moving human figure on the other hand depicted a 3D person, and might have induced a more vivid 3D percept. Finally, the complexity of the motion itself may differentiate the conditions, as more complex motion defining the object might provide better binding cues. In this case, the rectangle had in some sense the simplest motion (same linear trajectory for all the object points), whereas the human figure had more complex motion trajectories in space. We took these factors into account and further assessed LG's non-biological form from motion in Experiment 3.

## 5 Experiment 3

**Fig. 4:**
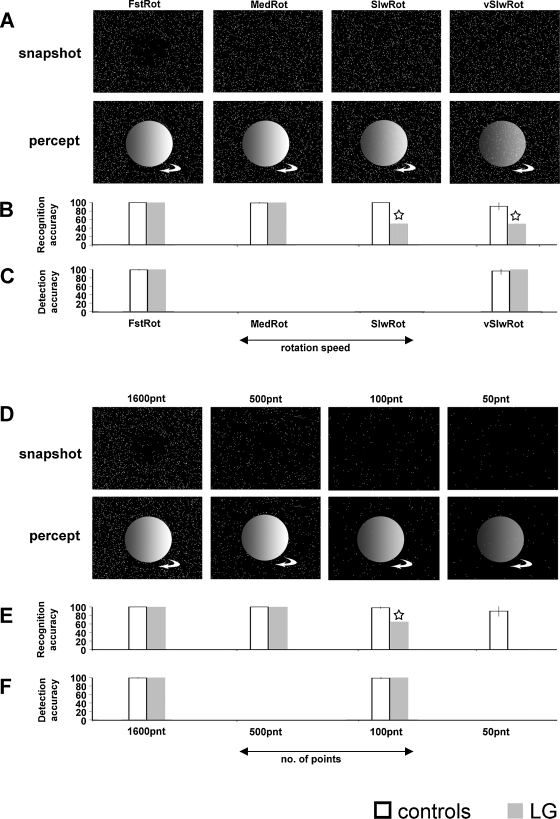
Experiment 3: paradigm and results. (A) Top: Snapshots of the random point/dot field stimuli of four conditions (FstRot, MedRot, SlwRot, and vSlwRot) varying the rotation speed of the object. Bottom: A depiction of the percept formed in normal observers by the local motion cues that are due to the object rotation. At very slow rotation speeds (vSlwRot condition and slower (data not shown)), the percept is reduced (depicted by a transparent object). Objects were spheres or cylinders rotating clockwise or anticlockwise (see Section 5.1 for further details). (B) Object recognition accuracy for LG (gray) and controls (white). An asterisk indicates significant reduction in performance in LG relative to controls (*p* < 0.005). Full details are provided in Table 2. (C) Object detection accuracy levels (same format as in B). (D–F) Same as in A–C, when the number of points defining the random point/dot field are being varied (conditions 1600pnt, 500pnt, 100pnt, and 50pnt).

This experiment sought to further assess LG's ability to identify and detect non-biological objects defined by motion. In the present experiment, we used more naturalistic, three-dimensional non-biological objects (spheres and cylinders) defined by motion cues. The motion was both characteristic of these objects (rotation/spin) and conveyed surface and three dimensional structure (Fig. 4A and D). Furthermore the local motions in space were more complex than translation.

The paradigm we used allowed the presentation of an object (sphere or cylinder) based only on the local motion vectors across the object (Singer & Sheinberg, 2008). An animated three-dimensional scene composed of a rotating object and a static background was rendered in real-time as a pattern of points. A global percept of the moving object (or a whole moving scene) emerged from the integration of the local motion vectors into a coherent moving shape. Thus, the perception of an object was based only on the motion vectors across the object. Since each point followed the trajectory of the underlying motion in the scene, only points located on the rotating object surface actually had local motion, while the points located “on the background” did not. Each static frame of the animation appeared to be a uniform random field of points (see Fig. 4A and D). By varying different parameters of this paradigm (number of points in the display and the rotation speed of the object) we were able to modulate task difficulty (see below).

### 5.1 Experiment 3: methods

#### 5.1.1 Experiment 3: stimuli

Throughout each experimental session, the display was composed of flickering white points that randomly appeared on a black screen (“formless dot field” random dot motion). Each point had a short lifetime (1.33 s, 80 frames at 60 frames/s) and the appearances of the points on the screen were not synchronized. When a rotating object trial began, the motion of a rotating object was embedded into the flickering point display. Flickering points that appeared in the location of the rotating object surface followed the local motion of the object's surface for the full extent of their lifetime (1.33 s). When a trial ended, all the points in the display had no local motion (i.e., each point appeared and stayed at the same location on the screen for its whole lifetime). The rotating object was either a 3D sphere or cylinder (Fig. 4A and D). Half of the trials were of a rotating sphere and half of a cylinder, and the order was determined randomly. The spinning object rotated around its north–south axis which was tilted 27° away from the screen's *y* axis plane (north end farther away, south end closer), similar to the Earth's tilt. The object rotation direction was determined randomly (clockwise or anticlockwise, 50% trials to each direction). The size of the sphere when viewed from 55 cm distance was 12 × 9.9° visual angle (width × height), the size of the cylinder was 8.2 × 9.4° (width × height) visual angle, and the point diameter was 0.16° visual angle. Screen resolution was 1024 × 768, refresh rate 60 Hz. Stimuli were presented using Matlab (Mathworks, Natick, MA, USA) and Psychophysics Toolbox 3 (Brainard, 1997; Pelli, 1997). The experimental stimuli were based on the FDFDemo and moglFDF functions provided with the Psychophysics Toolbox, which provides an OpenGL (Silicon Graphics Inc.) interface for Matlab.

#### 5.1.2 Experiment 3: tasks

##### 5.1.2.1 Object recognition

In the object recognition task, a rotating object appeared in every trial (a sphere or a cylinder) and participants’ task was to press a key once the object was recognized and then verbally indicate to the experimenter what the object was. The object rotated until the response was given without time restriction.

In four conditions, object rotation speed was parametrically set to 0.5 (FstRot), 0.0833 (MedRot), 0.0167 (SlwRot), or 0.0033 (vSlwRot) rotations/s, while the number of points composing the formless point field was constant (1600, see Fig. 4A–C). Four conditions (1600pnt, 500pnt, 100pnt, and 50pnt) included a parametric change to the number of points composing the formless point field (1600, 500, 100, or 50 respectively) while the rotation speed remained constant (0.5 rotations/s, see Fig. 4D–F). Note that the 1600pnt and FstRot conditions are identical (maximal rotation speed and maximal number of points).

##### 5.1.2.2 Object detection

The object detection sessions took place after the object recognition sessions. The stimuli in the object detection sessions were identical to those in the object recognition task in all aspects, except that the object was present in only half of the trials. In the other half of the trials, there was no local motion.

The rotating object (sphere or cylinder) appeared in 10 of the 20 trials and the participant had to press a key to indicate whether or not the object was present. After the key press, they had to verbally indicate by “yes” or “no” whether or not there was an object present. There was no time restriction for providing responses, and in the case of a present (rotating) object the object rotated until the key press. Before each object detection session of 20 trials with specific fixed parameters, participants were notified verbally by the experimenter about the approximate rotation speed of the objects in the session so that they would know what to expect for a ‘present object’ trial (e.g., “in this session the objects will be rotating really slowly”).

#### 5.1.3 Experiment 3: procedure

Participants viewed an example set of a few trials with 1600 points and rotation speed of 0.5 rotations/s, parameters that provided easy recognition of the objects even for LG (see Fig. 4A, conditions FstRot and 1600pnt) and reported verbally what object they saw on the screen. After these practice trials, each condition included a session of 20 trials with fixed parameters throughout the session (number of points, rotating speed). Participants’ verbal responses were recorded on paper and later digitized for further analysis. After the verbal response and once the participant was ready, another button was pressed to start the next trial.

#### 5.1.4 Experiment 3: data analysis

In this experiment for each experimental condition we compared LG's accuracy to those of the control participants and then determined whether LG's performance was significantly different from that of the controls’ as was done in Experiments 1 and 2.

### 5.2 Experiment 3: results and interim discussion

Fig. 4B and E depicts the object recognition results (see Table 2 for full performance details and statistical results). Object recognition was at ceiling for both LG and controls for the FstRot, MedRot, 1600pnt, and 500pnt conditions. However, when less object information was available in the display (slower rotation speed, 0.0167 (SlwRot) or 0.0033 (vSlwRot) rotations/s, or 100 points defining the object (100pnt)), LG's object recognition impairment became apparent. He reported not being able to do the task, and even without a time constraint, he claimed to be guessing. Even though in the 100pnt condition, his performance was better than chance (65%), this was significantly worse than controls’ recognition accuracy (98.13% ± 2.59 (S.D)).

In contrast to object recognition, LG's performance in the object detection task was perfect, indistinguishable from controls even when the displays contained sparse object information (vSlwRot and 100pnt conditions). He indicated verbally that he could see that “there is something” when an object was present (Fig. 4C and F, and Table 2 for full details), though he was unable to report which object it was. His normal detection ability might be accounted for by the local motion cues that the object induced. This motion could be easily detected without the need to integrate them into a coherent shape (Singer & Sheinberg, 2008).

In sum, consistent with the results observed in Experiment 2, LG displayed impaired recognition of form from non-biological motion. We thus verified that LG shows a dissociation between form from biological motion and from non-biological motion, possibly indicating there are distinct processes underlying biological motion perception.

## 6 fMRI connectivity analyses

Finally, we sought to examine whether LG's normal biological motion perception would be reflected in the functional integrity of the entire APS when viewing biological motion stimuli, despite the sparse and abnormal visual inputs from V2/V3 and ventral stream (Gilaie-Dotan et al., 2009).

In a recent neuroimaging study we localized LG's motion sensitive area V5/MT+ and found its activity to motion stimuli normal (Gilaie-Dotan et al., 2009). Here, we examined the functional connectivity of LG's right V5/MT+ to the rest of the brain using new analyses performed on the previously collected fMRI data.

### 6.1 fMRI connectivity: methods

LG was 21 years old when he participated in the fMRI experiments described below. Written informed consent to participate in these experiments was obtained prior to participation, according to the Tel-Aviv Sourasky Medical Center ethics committee that approved the experimental protocol.

#### 6.1.1 fMRI connectivity: procedure

The fMRI experiments are described below, and further details can be found in Supplementary Methods as well as in previous publications (Gilaie-Dotan et al., 2009; and see Avidan, Hasson, Malach, & Behrmann, 2005).

##### 6.1.1.1 V5/MT+ localizer

This experiment (as described earlier in Hasson, Harel, Levy, & Malach, 2003) sought to delineate motion-sensitive regions in the visual cortex (e.g., V5/MT+). The experiment comprised 2 conditions (“static” and “motion”) which were presented in blocks lasting 18 s, interleaved with 6-s fixation periods. Eight blocks of each condition were presented in the experiment. The stimuli for each condition consisted of low contrast rings (6% contrast, 2 cycles/° and a duty cycle = 0.2) surrounding the fixation point and forming a maximal visual angle of 16 × 16°. In the motion condition the rings either expanded or contracted (for 2 s in each direction of motion) at a rate of ∼6°/s, while in the static condition rings were displayed for 3 s each in a consecutive manner (hence not causing motion perception). LG was instructed to maintain fixation throughout the experiment. The experiment lasted 420 s.

##### 6.1.1.2 Movie clips experiment

In this experiment category-related video clips were presented in a block design. Each block lasted 15 s during which a clip was presented continuously. Based on their content, the clips formed four conditions: close-ups of people in various situations (“faces”), objects from different categories (tools, musical instruments, furniture, kitchenware, etc.; “objects”), navigation of the camera through open fields (“navigation”), and navigation through city buildings (“buildings”). The “faces” and “objects” conditions included biological motion of faces, hands and arms (manipulating the objects), while the other two conditions did not. Each condition was repeated 8 times with different clips at each repetition. The entire experiment lasted 12 min. Blocks were separated by a 6-s gray blank screen. The clips subtended a visual angle of 21° width × 17.3° height. LG was instructed to watch the movie-clips passively (see Gilaie-Dotan et al., 2009; and also Avidan et al., 2005).

#### 6.1.2 MRI data acquisition

Full details are provided in Supplementary Methods.

#### 6.1.3 fMRI data preprocessing and analysis

Full details are provided in Supplementary Methods. Briefly, preprocessing was applied to the functional data set of each experiment and the analysis was performed independently for each individual voxel. A general linear model (Friston et al., 1995) was fit to the time course of each voxel in the motion-selectivity experiment according to the experimental protocol.

##### 6.1.3.1 V5/MT+ definition

Motion-sensitive voxels were determined by contrasting the motion coefficient against the static coefficient. Right V5/MT+ ROI was determined for LG as the motion-sensitive region (motion > static) in the right middle temporal cortex, located ventrolaterally, just posterior to the meeting point of the inferior temporal sulcus and the lateral occipital sulcus in the vicinity of the middle occipital gyrus/sulcus based on a minimum cluster size of 6 functional voxels.

##### 6.1.3.2 Functional connectivity analyses

This analysis is based on correlating fMRI activations in LG's brain while viewing movie clips that included clips conveying biological motion. For every voxel independently, the correlation between its time course and the average time course of right V5/MT+ (that served as a seed to the correlation analysis), was obtained. These were subjected to a minimum cluster size of 8 voxels. Whole brain Bonferroni-corrected significant correlations (*r* >  = 0.377, *t*(235) = 6.24, *p*(corrected) < 0.0001) are described in Supplementary Methods and displayed in Fig. 1B.

### 6.2 fMRI connectivity: results

Of special interest here was whether the network of brain areas that are linked to biological motion processing (here referred to as the Action Perception System or APS) would display normal functional connectivity to V5/MT+. To address this, we used LG's right V5/MT+ activation time course while viewing the movie clips in a correlation analysis in order to examine which parts of LG's brain were functionally correlated with this activity (see Section 6.1). As expected, activity in LG's intermediate visual regions was not correlated with V5/MT+ activity (see Fig. 1B). However, surprisingly, despite a dominant component of LG's visual system that is abnormal, the regions comprising the APS exhibited activity that was significantly correlated with LG's V5/MT+ activity. The correlated regions included bilateral intraparietal sulcus, inferior frontal sulcus, and precentral sulcus, and the right superior temporal sulcus (see Fig. 1B).

## 7 General discussion

The extent to which biological motion perception relies upon processing of form by the ventral visual system is under debate (e.g., Blake & Shiffrar, 2007; Giese & Poggio, 2003). Using point-light displays conveying biological and non-biological form from motion, we investigated the ability of LG, a rare case of developmental visual-integrative agnosia with visual integration deficits (Gilaie-Dotan et al., 2009) to process and perceive biological and non-biological form from motion.

We found LG had normal perception of biological motion, even when tested with point-light stimuli, where the percept of a moving human figure emerges from spatially disconnected local motions of the point-lights, and integration of these into a coherent form is needed. In contrast, he was significantly deficient in processing non-biological form from motion. This pattern indicates that normal biological motion processing can be achieved independently from non-biological form from motion processing. Moreover, it emphasizes the necessity of proper ventral stream function for processing non-biological form from motion.

While we cannot completely rule out the possibility that LG has a subtle deficit in biological motion perception, we believe this possibility to be unlikely, because the paradigms we used here were sensitive enough to detect deficits in biological motion processing in other populations (e.g., in stroke patients, a notoriously heterogeneous sample (Saygin, 2007)). Moreover, LG demonstrated normal ability to process biological motion in two different experiments featuring different tasks (detection for Experiment 1 and direction determination for Experiment 2), plus in an additional variant of the detection task with a paradigm similar to Experiment 2 (data not shown). Since the functioning of LG's ventral visual stream is deficient, it stands to reason that his normal perception of biological motion relies on his normally functioning dorsal system. Consistent with this, we found normal activation and connectivity patterns in LG's motion sensitive lateral temporal area V5/MT+. LG might also be able to rely on higher brain areas that are part of the APS, such as the STS and premotor cortex (Grossman & Blake, 2001; Puce & Perrett, 2003; Saygin, 2007; Saygin et al., 2004), as functional connectivity in this network appeared normal in LG's brain. Thus, inputs to the APS from V5/MT+ can be sufficient for normal biological motion perception despite abnormal ventral stream function.

In contrast to his ability to perform well on biological motion tasks, LG showed impairments in processing non-biological form from motion. In Experiments 2 and 3, we found significant differences between LG and controls for non-biological object motion processing. These experiments also utilized different tasks in order to allow us to ascertain any deficits were not task-specific. Whereas the stimuli in Experiment 2 were two dimensional shapes that translated, LG still exhibited difficulty with non-biological object motion when we used three dimensional objects that carried out more object-characterizing movements (Singer & Sheinberg, 2008). Thus, LG's previously established deficits in form integration also extend to non-biological form from motion perception.

The finding that biological motion processing can dissociate from form integration does not imply that biological motion operates independently of form processing in the healthy brain. In fact, several studies suggest this is unlikely (Lange, Georg, & Lappe, 2006; Lange & Lappe, 2006; Vangeneugden et al., 2009). However, the present findings show that biological motion can be processed successfully even with compromised ventral stream integration. Perhaps specific for biological motion, the brain appears to be able to compensate for the absence of the normal contribution ventral system makes in perceiving form from motion. It is possible that the visual system may compute biological motion largely relying on a form-based template matching strategy (Lange et al., 2006; Lange & Lappe, 2006, 2007). In this framework, our data would indicate that these computations can be performed without reliance on ventral stream integration. Vangeneugden et al. (2009) recently discovered neurons in the STS that appear to be sensitive primarily to body posture rather than to the motion of biological motion stimuli (see also Jellema & Perrett, 2003; Oram & Perrett, 1996). Given LG's functional neuroanatomy, it is possible that the form processing resources in lateral temporal cortex can be sufficient for biological motion processing. More generally, biological and non-biological form from motion processing may rely differentially on templates that are computed or stored in distinct brain areas (e.g., lateral temporal vs. ventral temporal areas).

Taken together, these data indicate that normal inputs from V5/MT+ can be sufficient for the APS to process biological motion. In high-order ventral cortex, form inputs arriving from retinotopic regions are supported by motion cues from V5/MT+, to create a coherent percept (Felleman & Van Essen, 1991; Grill-Spector, Kushnir, Edelman, Itzchak, & Malach, 1998; Singer & Sheinberg, 2010; Ungerleider & Desimone, 1986) but LG's case suggests that input from V5/MT+ cannot completely overcome the lack of inputs from intermediate retinotopic cortex.

In conclusion, the present data demonstrate that although form from motion perception from point-light displays requires form integration, it is possible to process biological form from motion even if ventral stream integration is deficient. Our findings extend prior work showing biological motion can dissociate from other kinds of motion perception (e.g., McLeod, Dittrich, Driver, Perrett, & Zihl, 1996; Saygin, 2007; Vaina, Lemay, Bienfang, Choi, & Nakayama, 1990). In addition, we show that it can also dissociate from form integration. It is therefore possible that there are multiple (and flexible) substrates for biological motion processing, possibly due to the evolutionary importance of the domain.

## Figures and Tables

**Table 1:** Schematic description of LG's perception and brain function of motion and form.

	Motion	Form
Behaviour	*Normal* (self report)	*Deficient*
	“I recognize people by the way they walk”	e.g., Hooper Visual Organization Test: *high probability of impairment*

Brain	*Normal* V5/MT+ motion sensitivity	*Abnormal* visual hierarchy activations (from intermediate visual regions)
	*Normal* V5/MT+ functional connectivity to ASP (see Fig. 1)	*Abnormal* form sensitivity in ventral cortex (see Fig. 1)

**Table 2:** Experiment 3: detailed results and statistical analysis. Results in bold indicate conditions that LG performed significantly below controls.

No. of points	Rotation/s	Condition name	Task	Accuracy			*t*(7)_Crawford_	*p*_Crawford_	*t*(7)_Crawford_	*p*_Corballis_
				Controls (mean)	Controls (S.D.)	LG				
1600	0.5	FstRot/1600pnt	Recognize	100	0	100	0	1	0	1
			*Detect*	*99.38*	*1.77*	*100*	*0.33*	*0.75*	*0.35*	*0.73*
	0.0833	MedRot	Recognize	99.38	1.77	100	0.33	0.75	0.35	0.73
	0.0167	SlwRot	Recognize	100	0	**50**	**−4714**	**5 × 10**^**−24**^	**−5000**	**4 × 10**^**−24**^
	0.0033	vSlwRot	Recognize	91.25	9.54	**50**	**−4.075**	**0.0047**	**−4.322**	**0.0035**
			*Detect*	*96.25*	*8.76*	*100*	*0.40*	*0.70*	*0.428*	*0.68*

500	0.5	500pnt	Recognize	100	0	100	0	1	0	1
100		100pnt	Recognize	98.13	2.59	**65**	**−12.07**	**6.12 × 10**^**−6**^	**−12.8**	**4.12 × 10**^**−6**^
			*Detect*	*98.75*	*2.31*	*100*	*0.51*	*0.63*	*0.54*	*0.61*
50		50pnt	Recognize	90	12.82					
