# Supplementary material for: Normal form from biological motion despite impaired ventral stream function
Source: Neuropsychologia. 2011 Apr;49(5):1033–43. doi: 10.1016/j.neuropsychologia.2011.01.009 (PMC3083513; doi:10.1016/j.neuropsychologia.2011.01.009)
Supplement: Supplementary file 1 [file mmc1.doc]

**Supplementary Material**

**Normal form from biological motion despite impaired ventral stream function**

Gilaie-Dotan S, Bentin S, Harel M, Rees G & Saygin AP

# Supplementary Methods

#### *MRI Data Acquisition*

LG was scanned in a 1.5 T Signa Horizon LX 8.25 GE scanner (GE Healthcare, Piscataway, NJ). Blood oxygenation level-dependent (BOLD) contrast images were obtained using a gradient-echo echo-planar imaging (EPI) sequence with time repetition = 3000 ms, time echo = 55 ms, flip angle = 90º, field of view 24 * 24 cm^2^, matrix size 80 * 80. The scanned volume included 24-25 nearly axial slices for LG in order to cover his entire cortex (via a standard head coil). Slice thickness was 4 mm with 1 mm gap with an in-plane resolution of 3 * 3 mm^2^.

A whole-brain spoiled gradient (SPGR) sequence was also acquired for LG (field of view 240 * 240 mm^2^, matrix size 256 * 256, slice thickness 1.2 mm, 124 axial slices) to allow accurate cortical segmentation, reconstruction, and volume-based statistical analysis (slices from this scan are provided in Fig. 1C). In addition, high-resolution (1.1 x 1.1 mm^2^) T1-weighted anatomic images of the same orientation and thickness as the EPI slices were also acquired to facilitate the incorporation of the functional data into the 3D Talairach space (Talairach & Tournoux, 1988). The cortical surface was reconstructed from the 3D SPGR scan. The procedure included segmentation of the white matter using a grow region function, the smooth covering of a sphere around the segmented region, and the expansion of the reconstructed white matter into the gray matter. The surface of each hemisphere was then unfolded, cut along the calcarine sulcus and additional predefined anatomical landmarks on the medial side, and flattened.

#### *fMRI Data Preprocessing and Analysis*

fMRI data were analyzed with the BrainVoyager software package (R. Goebel, Brain Innovation, Maastricht, The Netherlands) plus additional in-house software. The first two or three images of each functional scan were discarded. The functional images were superimposed on 2D anatomic images and incorporated into the 3D data sets (see structural MRI above) through trilinear interpolation. The complete data set was transformed into Talairach space (Talairach & Tournoux, 1988). Preprocessing of functional scans included 3D motion correction, slice scan time correction, linear trend removal and filtering out of low frequencies up to 10 cycles per experiment.

A general linear model (Friston et al., 1995) was fit separately to the time course of each individual voxel in the motion-selectivity experiment according to the experimental protocol. The model coefficients for each voxel were determined so that the error term between the model’s prediction and the measured voxel time course is minimized (Least Squares method). The analysis was performed independently for each individual voxel. A t-test between coefficients of different conditions was applied to determine a voxel’s activation pattern, taking into account the error term. The voxel’s P value was determined as the P corresponding to the resulting t value of the t-test.

#### *fMRI Functional Connectivity Analysis*

Correlation significance for each voxel was determined by$t=\frac{r}{\sqrt{(1-r^{2})/235})}$, where *r* was the correlation between a voxel’s time course and right MT’s time course, 235 was the degrees of freedom for correlation between time courses of 237 time points. To achieve Bonferroni corrected significance, the *p* value corresponding to the *t* statistics was divided by the size of the sample size, i.e. number of voxels in LG’s brain.

# Supplementary Figures Legends

These supplementary figures are animated gifs and should be viewed with a web browser, Quicktime, or Media Player. Other softwares might also support viewing the motion embedded in the animated gifs. Note that presentation speed might vary according to the viewing software and thus may not represent the actual speed of the motion as appeared in the experiments.

**Supplementary Figure 1**

A schematic demonstration of the setup of Experiment 1. On the left side is a biological motion point light display (PLD) depicting an underarm throwing as in bowling, and on the right side a spatially scrambled version of this animation is presented. Both of these are embedded in additional noise points. Participants had to determine the side of the biological motion (right or left). See main text for further details.

**Supplementary Figure 2**

Biological motion animation of throwing, which is one of the seven actions used in Experiment 1.

**Supplementary Figure 3**

Biological motion animation of a walking upright human figure, which is one of the seven actions used in Experiment 1. This animation was also used in Experiment 2 in the BM condition.

# Supplementary References

Friston, K. J., Holmes, A. P., Poline, J. B., Grasby, P. J., Williams, S. C., Frackowiak, R. S., et al. (1995). Analysis of fMRI time-series revisited. *Neuroimage, 2*(1), 45-53.

Talairach, J., & Tournoux, P. (1988). *Co-Planar Stereotaxic Atlas of the Human Brain*. New York: Thieme Medical Publishers.
