# Supplementary figures and images for: Normal form from biological motion despite impaired ventral stream function
Source: Neuropsychologia. 2011 Apr;49(5):1033–43. doi: 10.1016/j.neuropsychologia.2011.01.009 (PMC3083513; doi:10.1016/j.neuropsychologia.2011.01.009)

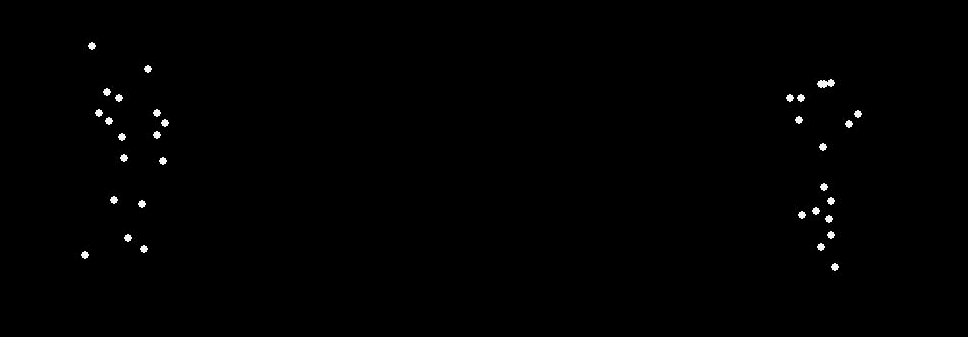

Supplement: Supplementary file 2 [file mmc2.gif]

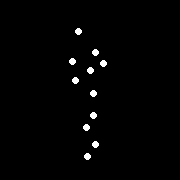

Supplement: Supplementary file 3 [file mmc3.gif]

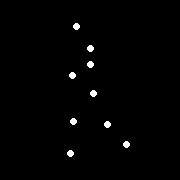

Supplement: Supplementary file 4 [file mmc4.gif]
